# Synthesis of New D–π–A Phenothiazine-Based Fluorescent Dyes: Aggregation Induced Emission and Antibacterial Activity

**DOI:** 10.1007/s10895-024-03708-7

**Published:** 2024-05-09

**Authors:** Mervat S. El-Sedik, Mahmoud Basseem I. Mohamed, Mohamed S. Abdel-Aziz, Tarek S. Aysha

**Affiliations:** 1 Dyeing, Printing and Textile Auxiliaries Department, Textile Research and Technology Institute, National Research Centre, 33 EL Buhouth St., Dokki, Giza, 12622 Egypt; 2 Chemistry Department, Faculty of Science, Al-Azhar University, P.O. 11884, Nasr City, Cairo, Egypt; 3 Microbial Chemistry Department, Biotechnology Research Institute, National Research Centre, 33 EL Buhouth St., Dokki, Giza, 12622 Egypt

**Keywords:** Phenothiazine, Sulfa-drugs, Fluorescent dyes, Aggregation-induced emission, Solvatochromic, Antibacterial affinity

## Abstract

Highly solid-state fluorescent dyes based on phenothiazine bearing sulfa-drug derivatives were successfully prepared and fully characterized by NMR, mass spectra, and elemental analysis. The prepared phenothiazine dyes bearing sulfadiazine and sulfathiazole 4-(((10-hexyl-10 H-phenothiazin-3-yl)methylene)amino)-*N*-(pyrimidin-2yl) benzenesulfonamide (**PTZ-1**) and 4-(((10-hexyl-10 H-phenothiazin-3-yl) methylene) amino)-*N*-(thiazol-2-yl)benzenesulfonamide (**PTZ-2**), showed strong emission in polycrystalline form, and significant emission in solution was observed. The quantum yield of the prepared dyes varied and decreased by increasing the solvent polarity, with the maximum recorded value being 0.63 and 0.6 in dioxane. Aggregation-induced emission (AIE) and the effect of the solvent polarity on absorption and emission spectra were investigated. The dyeing application of polyester fabrics using the prepared phenothiazine-based dyes was studied, showing very good affinity to dyed fabrics. The antibacterial affinity against gram-positive and gram-negative bacteria for the dye powder as well as the dyed PET fabric was investigated, with **PTZ-2** showing better affinity against bacteria compared to **PTZ-1**. This multifunctional property highlights the potential uses of **PTZ-1** and **PTZ-2** for advanced applications in biomedicine and optoelectronics.

## Introduction

The fluorescence characteristics of the dyes can be observed based on their ability to absorb electromagnetic radiation and emit the absorbed energy. The emission of the dye in powder form is one of the interesting features of organic dyes for a wide range of applications [1–8]. The use of fluorescent dyes in textile dyeing can brighten the color and create textile products that are highly visible when using certain materials [6, 9, 10].

The impressive optical effect of fluorescence is of interest not only for marking inks but also for safety wear and signage, as well as for security printing and leak proofing. The chemistry and physics of fluorescent dyes are well developed, with many applications in an expanding field such as optical whitening agents for paper, textiles and various other white or bright materials [7, 11–14], laser dyes and organic light emitting diodes (OLEDs) [15, 16]. The application of fluorescent dyes in health-related field is considered one of the most important functional applications and researchers pay more attention to these topics. Since many applications in vitro or in vivo cannot be seen directly and must be viewed with the use of imaging equipment, the fluorescence change of some organic dyes could be very important techniques for this kind of application [17]. One of the biggest public health concerns now is bacteria, which has been linked to many hospital-acquired diseases [18]. There was a major advancement in biochemistry thanks to the green fluorescent protein. The understanding of the interaction of light with matter is advantageous for applications and future advances of fluorescent materials. Consequently, in both science and technology, the interaction of light with matter is becoming increasingly important. Aggregation-induced emission (AIE) is one of the interesting phenomena that occur when molecules aggregate and emit more fluorescence than when they are isolated [19–26]. Additionally, the AIE effect allows most fluorophores to overcome limits created by ACQ (aggregation-caused quenching) that takes place due to extremely concentrated liquids or solid states, mainly aqueous solutions. Moreover, the ACQ impact can be effectively reduced by the AIE behavior, which opens the door for the use of organic emitters in bioimaging or optoelectronic devices [27, 28]. Fascinatingly, the aggregation induced emission (AIE) phenomena are ultimately generated due to the limited intramolecular motions [29, 30].

The phenothiazine unit is a nonplanar butterfly-shaped tricyclic heteroarene that is rich in electrons due to the presence of sulfur and nitrogen atoms. Additionally, phenothiazine has a significant electron-donating ability, so it was frequently utilized as an active component in push-pull chromophores. According to earlier research, 3-position mono-substituted D-A phenothiazine derivatives have outstanding force response [31]. . Phenothiazine based fluorescence dyes have been used as efficient materials in many applications such as colorimetric chemosensors and fluorescence probes for monitoring ions [32], in drug delivery applications, dye sensitized solar cells (DSSCs) [33–38], pharmaceuticals and electrochemistry [39, 40].

Sulfa drugs and their metal complexes have a wide range of uses, including as diuretics, glaucoma treatments, and epilepsy medications. Sulfa-drugs have significant biological activity; for instance, their mode of action is based on the antagonistic competition between PABA (*p*-aminobenzoic acid) and sulfanilamide [41–43].

Microorganisms have a greater proclivity for causing damage to textile materials including microbes, algae, fungi, viruses, and bacteria. In this case, the fabrics act as active agents in the spreading of microbes and as a medium for microbial growth as well [44, 45]. Such proclivity generally causes stains, color fading, deterioration of the product, skin infection, allergic diseases and an annoying odor on the wearer. Hence, due to the high efficiency of sulfa-drugs against bacteria and the high emission characteristics of phenothiazine derivatives this work presents a new analogue of fluorescence phenothiazine bearing sulphadiazine and sulphathiazole-based dyes. The spectral properties and solvatochromic effect on absorption and emission spectra were investigated, aggregation-induced emission, antibacterial efficiency against gram positive and gram-negative bacteria as well as the dyeing applications on polyester fabrics as highly emissive dyes were studied.

## Experimental

### Materials and Apparatus

Phenothiazine (98%), 1-bromo hexane, sulfathiazole and sulfadiazine were purchased from Sigma-Aldrich Germany. The solvents used in this study were analytical grade and used without any further purifications such as DMF, acetone, ethanol, methanol, 1,4-dioxane, acetonitrile and n-hexane. Thin layer chromatography (TLC) (Merck, DC Kiesel gel 60 F254) plates were used for monitoring the consumptions of reactant and the forming of the new product. NMR spectra was recorded on a Bruker DMX-400 spectrometer operating at 400, 101 MHz using DMSO-*d*_*6*_ as solvent. LC/MS with ESI electrospray ionization source (positive ion mode) was investigated using a XEVO TQD triple quadrupole mass spectrometer. UV/vis absorption spectra were measured on a Shimadzu UV spectrophotometer and emission spectra was investigated using a JASCO fluorimeter (8300).

### Synthetic Routes of Fluorescent Dyes PTZ1 and PTZ2

#### Synthesis of 10-hexylphenothiazine (Ia)

In 250 mL two-necked round flask connected with condenser phenothiazine (47.6 mmol, 11.64 g), potassium hydroxide (142.8 mmol, 8 g) was added and stirred with 150 mL of *N*-Methyl-2-pyrrolidone (NMP). Then hexyl iodide (71.6 mmol, 15.2 gm) and 1 gm of potassium iodide were added to the mixture and the mixture was allowed to stirring for further 15 h at room temperature. Then the product was extracted using ethyl acetate as a colorless liquid with 90% yield. The characterization data such as NMR and mass spectra was agree with the previously published procedure [46].

#### Synthesis of 10-hexyl-10 H-phenothiazine-3-carbaldehyde (Ib)

10-hexyl-10 H-phenothiazine-3-carbaldehyde (**Ib**) was prepared by the same procedure described previously [47–50]. In three-nicked round flask connected with dropping funnel, condenser and thermometer, *N, N*-dimethylformamide (DMF) (100 mmol, 7.3 g) was add and cooled down in ice bath at 0 °C, phosphorus oxychloride (200 mmol, 30.6 g) was added dropwise during 1 h and the solution was allowed to warm to room temperature. Then, a solution of **1a** (20 mmol, 5.7 g) in dichloromethane (50 mL) was added. The mixture was allowed to heat up to 80 °C and stirred for a further 12 h. The reaction mixture was added to 300 mL of ice water and stirred for 5 mints then neutralized with sodium carbonate to pH 6–7 and continued stirring for further 30 min and the product **Ib** was extracted by ethyl acetate. The formed dark extracted product was purified by column chromatography on silica gel using n-hexane: acetone (10:1) as eluent to obtain (5.0 g, yield: 80%).

**Mass: m/z (%)**: 312.25 (100%).

#### Synthesis of 4-(((10-hexyl-10 H-phenothiazin-3-yl)Methylene)Amino)-*N*-(Pyrimidin-2-yl)Benzenesulfonamide (PTZ-1)

1.61 mmol, 0.5 g of compound (**Ib**), 1.61 mmol, 0.401 g of sulfadiazine and 50 mL of methanol were added to 100 ml round flask. The mixture was refluxed for 35 h with vigorous stirring with addition of catalytic amount (3–5 drops) of acetic acid. Then the mixture was cooled down and the yellow precipitate was collected by filtration and washed twice with ethanol and water then dried in oven at 60 °C forming 0.55 g of **PTZ-1 (dye 1)**, 58% yield.

**Anal. calcd (%)** for C_29_H_29_N_5_O_2_S_2_: C, 64.06; H, 5.38; N, 12.88; S, 11.79;

##### Found

C, 64.35; H, 5.42; N, 12.92; O, 5.92; S, 11.55.

**Mass: m/z (%)**: 544.18 (100%).

##### ^1^H-NMR (400 MHz, DMSO-*d*_*6*_, δ ppm)

0.79 (d, *J =* 5.8 Hz, 3 H), 1.21 (d, *J =* 2.0 Hz, 4 H), 1.36 (d, *J =* 4.9 Hz, 4 H), 3.89 (d, *J =* 6.0 Hz, 2 H), 6.95 (dd, *J =* 7.8, 2.2 Hz, 1 H), 7.04 (d, *J =* 7.3 Hz, 2 H), 7.16–7.06 (m, 2 H), 7.19 (dd, *J =* 7.7, 2.1 Hz, 1 H), 7.31 (t, *J =* 6.4 Hz, 2 H), 7.63 (d, *J =* 4.4 Hz, 1 H), 7.75–7.67 (m, 1 H), 7.96 (t, *J =* 6.3 Hz, 2 H), 8.45 (d, *J =* 5.9 Hz, 1 H), 8.49 (dd, *J =* 5.6, 5.0 Hz, 2 H), 11.75 (s, 1 H).

##### ^13^C-NMR (126 MHz, DMSO-*d*_*6*_, δ ppm)

13.14, 22.09, 26.13, 27.03, 31.35, 48.09, 113.89, 115.73, 116.10, 118.09, 121.00, 123.08, 124.97, 125.87, 126.11, 127.57, 128.01, 128.29, 128.39, 130.11, 131.68, 143.15, 144.08, 150.60, 152.07, 155.15, 161.59, 191.11.

#### Synthesis of 4-(((10-hexyl-10 H-phenothiazin-3-yl)Methylene)Amino)-*N*-(Thiazol-2-yl)Benzenesulfonamide (PTZ-2)

1.61 mmol, 0.5 g of compound (**Ib**), 1.61 mmol, 0.409 g of sulfathiazole and 50 mL of methanol were added to 100 ml round flask. The mixture was refluxed for 35 h with vigorous stirring with addition of catalytic amount (3–5 drops) of acetic acid. Then the mixture was cooled down and the yellow precipitate was collected by filtration and washed twice with ethanol and water then dried in oven at 60 °C forming 0.57 g of **PTZ-2 (dye 2)**, 65% yield.

**Anal. calcd (%)** for C_28_H_28_N_4_O_2_S_3_: C, 61.29; H, 5.14; N, 10.21; S, 17.53;

##### Found

C, 61.52; H, 5.12; N, 10.29; S, 17.62.

**Mass: m/z (%)**: 549.14.

##### ^1^H NMR (400 MHz, DMSO-*d*_*6*_, δ ppm)

0.78 (t, *J =* 6.6 Hz, 3 H), 1.31–1.14 (m, 4 H), 1.36 (d, *J =* 6.4 Hz, 4 H), 3.89 (t, *J =* 6.8 Hz, 2 H), 6.80 (d, *J =* 4.6 Hz, 1 H), 6.94 (t, *J =* 7.4 Hz, 1 H), 7.02 (d, *J =* 8.1 Hz, 1 H), 7.09 (d, *J =* 8.6 Hz, 1 H), 7.12 (d, *J =* 7.6 Hz, 1 H), 7.23 (d, *J =* 4.6 Hz, 1 H), 7.21–7.15 (m, 1 H), 7.28 (d, *J =* 8.5 Hz, 2 H), 7.63 (d, *J =* 1.6 Hz, 1 H), 7.70 (dd, *J =* 8.5, 1.6 Hz, 1 H), 7.78 (d, *J =* 8.5 Hz, 2 H), 8.44 (s, 1 H), 12.69 (s, 1 H).

##### ^13^C NMR (126 MHz, DMSO-*d*_*6*_, δ, ppm)

14.32, 22.54, 26.20, 26.62, 31.28, 47.52, 113.00, 116.08, 117.07, 121.77, 123.13, 124.11, 124.18, 127.64, 127.78, 128.26, 128.29, 128.47, 130.65, 131.38, 143.56, 144.09, 150.62, 152.77, 155.25, 161.52, 191.05.

### Spectral Measurements

Absorption and emission spectra were measured for the prepared dyes (**PTZ-1** and **PTZ-2**) in solution using a quartz cuvette with 1 cm path length. The solution was prepared by dissolving 20 µmol of dye in aprotic polar and non-polar solvents and absorption spectra were recorded using spectrophotometer. The relative fluorescence quantum yield of **PTZ-1** and **PTZ-2** was determined in different polar and nonpolar solvents using anthracene in ethanol with a concentration of 10^− 5^ mol/L and a quantum yield (Φ_s_ = 0.27) as the reference compound. The fluorescence quantum yield was calculated according to Eq. 1:

1 $${\phi _{\text{x}}} = \frac{{{{\text{A}}_{\text{s}}} \times {\text{}}{{\text{F}}_{\text{x}}} \times {\text{}}\eta _{\text{x}}^{\text{2}} \times {\text{}}{\phi _{\text{s}}}}}{{{{\text{A}}_{\text{x}}} \times {\text{}}{{\text{F}}_{\text{s}}} \times {\text{}}\eta _{\text{s}}^{\text{2}}}}$$

Where, A_s_ and A_x_ are the absorbance at the excitation wavelength of the standard and the sample of unknown, respectively. F_s_ and F_x_ are the areas under the fluorescence curve of the reference and the sample of unknown, respectively. η_s_ and η_x_ are the refractive indices of the standard and the sample of unknown, respectively [51].

Aggregation-induced emission (AIE) spectra were recorded at a range of water to dioxane concentrations (0–90% water) and the effect of sodium chloride (NaCl) was carried out using a certain concentrations from 0.01 to 1%, which prepared by dissolving NaCl (1 g/10 mL of H_2_O), the solution was diluted and utilized in all the experiments with a consistent volume of 1 mL in 10 mL of dioxane. The emission spectra were investigated in solution and in powder form using a special unit for powder holder and the effect of polarity of the solvents was studied in the case of solutions.

### Antibacterial Investigation

A volume of 2 mL of DMSO was used to dissolve 5 mg of each sample. The test strain bacteria utilized were *Staphylococcus aureus* ATCC 6538-P (G + ve), *Escherichia coli* ATCC 25,933 (G-ve), and *Candida albicans* ATCC 10,231 (yeast).

The antimicrobial activity of the prepared dyes **PTZ-1** and **PTZ-2** was assessed by the cup agar diffusion procedure. Bacterial and yeast test microbes were inoculated on nutrient agar medium plates seeded with 0.1 mL of 10^5^-10^6^ cells/mL while the fungal test strain was cultivated on plates with potato dextrose agar medium that seeded with 0.1 mL (106 cells/mL) of the fungal inoculum. 100 µl from each sample were distributed in holes developed in each inoculated plate. The plates were kept at low temperature (4 °C) for 2–4 h to allow maximum diffusion. The plates were then incubated at 37 °C for 24 h for bacteria and at 30 °C for 48 h for the fungus in upright position to allow maximum growth of the organisms. The antimicrobial activity of the test agent was measured by detecting the diameter of the inhibition zone expressed in millimeters (mm). The experiment was carried out multiple time, and the readings were recorded.

The antibacterial behavior of the dyed fabrics was determined by measuring colony forming units (CFU). The dyed polyester fabrics with dyes **PTZ-1** and **PTZ-2** were evaluated for their antibacterial activities using (CFU) procedure. The test bacterial strains icluded Staphylococcus aureus ATCC 6538-P (G + ve bacterium) and Escherichia coli ATCC 25,933 (G-ve bacterium). Bacterial stocks (100 µl of stock with a CFU value of approximately 108) were inoculated into a 20 mL of freshly prepared liquid nutrient broth containing 5 g/L peptone and 3 g/L beef extract at pH 6.8 in 100 mL volume of Erlenmeyer flasks and incubated for 24 h. Fabrics each of about 250 mg were added to the bacterial inoculated medium in 100 mL conical flasks each has 10 ml culture medium and inoculated by 20 µL of bacterial inoculums leaving the control (inoculated flasks without samples). After 24 h incubation at 37 °C, a serial dilution from each sample-containing culture and the controls has been done (10-1-10-4). The microbial inhibition was determined by counting the colony forming units (CFU) by inoculating petri-dishes containing solidified nutrient agar medium with 50 µL from each dilution and calculating the reduction growth rate (R) for treated samples in relation to control (untreated) according to the Eq. 2.

2 $${\text{R}}\left( {\text{\% }} \right) = {\text{B}} - \frac{{\text{A}}}{{\text{B}}} \times 100$$

Where A is CFU/mL for treated sample after 16 h incubation and B is CFU/mL for untreated sample after the same period of incubation time [52]. The optical density of the incubated liquid culture medium was recorded at 600 nm. The greater the growth, the higher the turbidity, and the optical density figure was therefore directly proportional to the number of bacteria in the medium.

### Dyeing Application of the Prepared Dyes on Polyester Fabrics

The dye dispersion was initially prepared by well milling the dye powder and the required amount of the dye was mixed with anionic dispersing agent (Sera Gal P-LP; DyStar, Egypt) in a 1:1 ratio with 0.5 mL of *N*,*N*-dimethylformamide. The resulting mixture was used and mixed with 2 mL of water. This mixture was added to the dyebath.

#### Dyeing Procedure

The previously prepared dye dispersions **PTZ-1** and **PTZ-2** with varying dye concentrations (0.5, 1, 2, and 3% omf) were used to carry out the dyeing operation. The bath’s pH was adjusted to 4–5. The polyester fabrics were dyed by increasing the temperature to 130 °C at a rate of 2.5 °C per minute and the dyeing process was continuous for 60 mints. The samples that had been dyed were then taken out, thoroughly rinsed with water, washed off with 2 g/L nonionic detergent (Sera Wash M-RK; DyStar) at a liquor ratio of 50:1 and at 60 °C for 15 min., rinsed in cold running water and left to dry in the open air.

The light reflectance approach using Kubelka-Munk according to the Eq. 3 was used to measure the relative color strength (K/S) of colored materials [53]. The reflectance (R) of the dyed fabrics was measured on Shimadzu UV/Vis spectrophotometer.

3 $${\text{K}}/{\text{S}} = \frac{{{{(1 - {\text{R}})}^2}}}{{2{\text{R}}}}$$

Where, R = Decimal fraction of the reflection of the dyed fabric, K = Absorption coefficient, and S = scattering coefficient.

The dye exhaustion (*%E*) on PET fabrics was measured and calculated by spectrophotometric method using Eq. 4.

4 $${\text{\% E}} = \frac{{{{\text{A}}_1} - {{\text{A}}_2}}}{{{{\text{A}}_1}}} \times 100$$

Where A_1_ and A_2_ are the absorptions of the dyebath, dissolved in acetone, before and after dyeing.

#### Fastness Measurements

According to ISO standard test procedures, a specimen of dyed polyester fabrics with a 1% omf depth of shade was evaluated. According to ISO 105-C06 B2S, the wash fastness test was evaluated at 50 °C for 30 min. with a liquor ratio of 50:1 using 4 g/L ECE detergent, 1 g/L sodium perborate, and 25 steel balls [54]. The rubbing fastness was investigated according to the stander method BS EN ISO 105 × 12 for determining the resistance of the color of textiles [55]. According to ISO 105-P01, the sublimation fastness test was performed with a fixometer at 180 and 210 °C [55]. In compliance with ISO 105-B02, a xenon arc lamp test was used to evaluate the light fastness test [56].

## Results and Discussions

### Synthesis of Intermediates

**Scheme 1:**
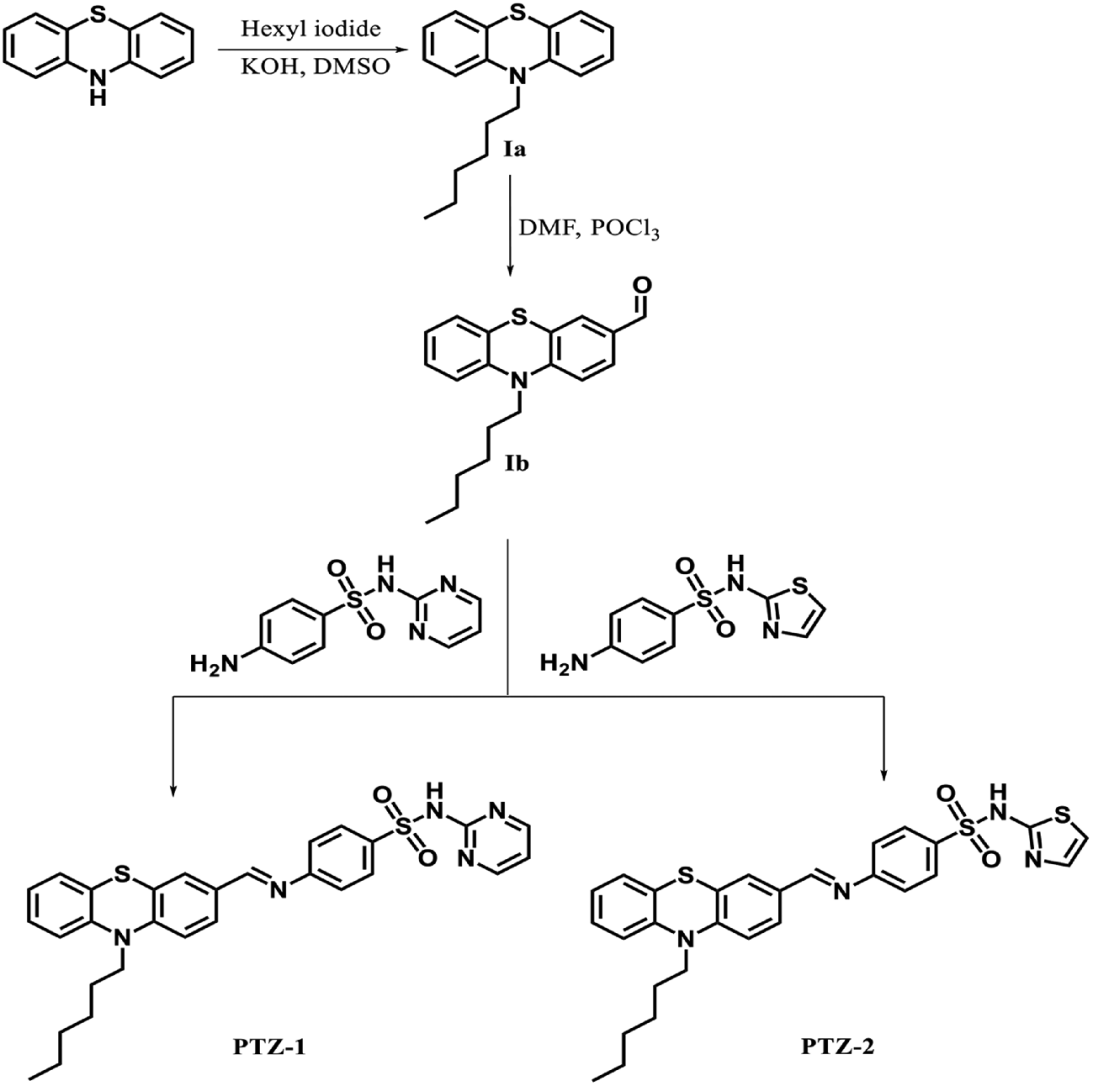
The synthetic routes for **PTZ-1**, and **PTZ-2**

The synthetic procedure of **PTZ-1**(dye 1) and **PTZ-2**(dye 2) was summarized in Scheme 1. Starting with the direct alkylation of phenothiazine to form 10-hexyl-10 H-phenothiazine (**Ia**) with an excellent synthetic yield of 90%, Vilsmeier formylation of **Ia** with phosphorus oxychloride and *N*,*N*-dimethylformamide in 1,2-dichloroethane provides aldehyde **Ib** in a regioselective approach with an 80% yield as bright yellow crystals.

The ^1^HNMR and ^13^CNMR studies of the synthesized dyes show significant peaks of aliphatic and aromatic protons and carbons as shown in supplementary file. The aliphatic protons corresponding to the methylene and methyl groups appear in the range of 0.78–3.89 δ while the aromatic protons appear in the range of 6.80–8.45 δ. The sulfonamide NH proton appears at 11.75 δ for **PTZ-1** and 12.69 δ for **PTZ-2**.

### Absorption and Fluorescence Study of PTZ-1, and PTZ-2

The spectral properties of the synthesized dyes **PTZ-1**, and **PTZ-2** were investigated, and the spectral data are summarized in Table 1.

**Table 1:** Photophysical properties of the synthesized dyes in different solvent, powder form and on dyed fabrics

Solvent	Dyes									
	PTZ-1					PTZ-2				
	λ_max/abs_ (nm)	Abs.	λ_max/em_ (nm)	Stokes shift (nm)	Quantum yieldΦ	λ_max/abs_ (nm)	Abs.	λ_max/em_ (nm)	Stokes shift (nm)	Quantum yieldΦ
EtOH	396	0.189	607	211	0.20	395	0.174	600	205	0.23
Acetonitrile	391	0.328	600	209	0.32	392	0.305	601	209	0.30
DMF	398	0.144	575	177	0.43	399	0.177	585	186	0.50
DCM	400	0.144	600	200	0.45	397	0.144	550	153	0.40
Dioxane	395	0.294	567	172	0.62	391	0.284	561	170	0.63
Toluene	398	0.137	555	157	0.37	393	0.064	547	154	0.30
Solid	--	--	558	--	--	--	--	538	--	--
Fiber	--	--	563	--	--	--	--	532	--	--

**Fig. 1:**
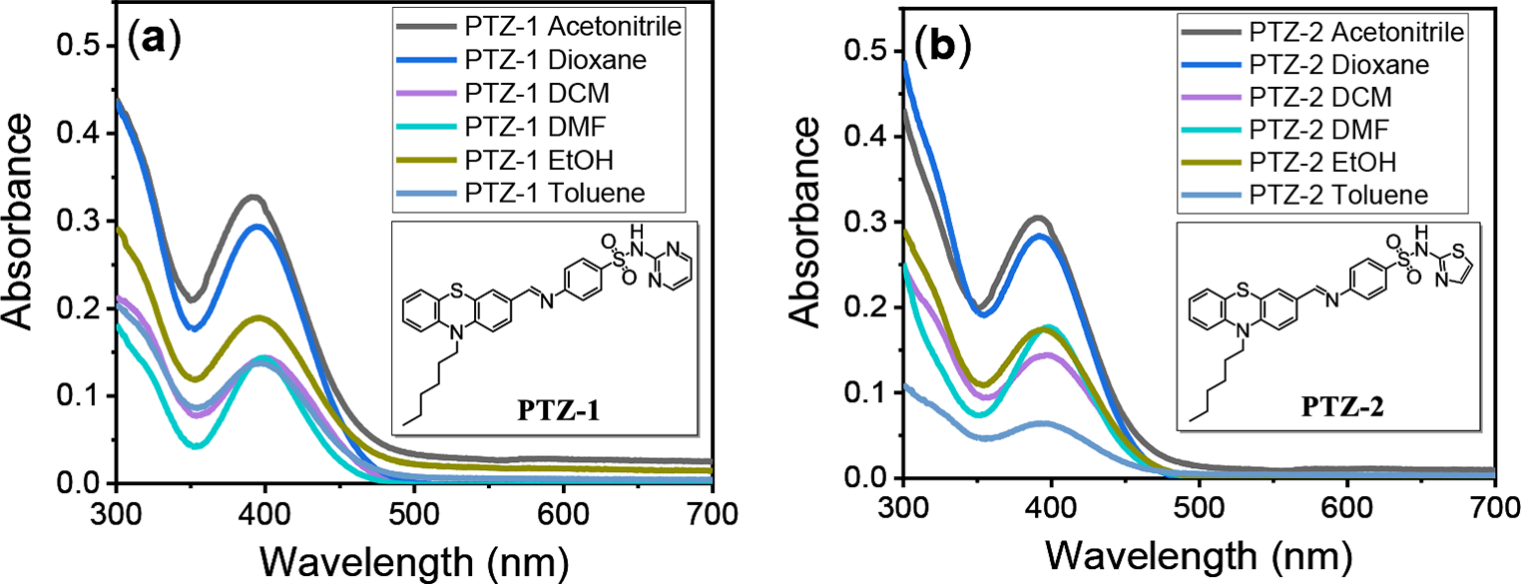
Absorption spectra of 10 µmole (**a**) **PTZ-1** and (**b**) **PTZ-2** in different solvents

**Fig. 2:**
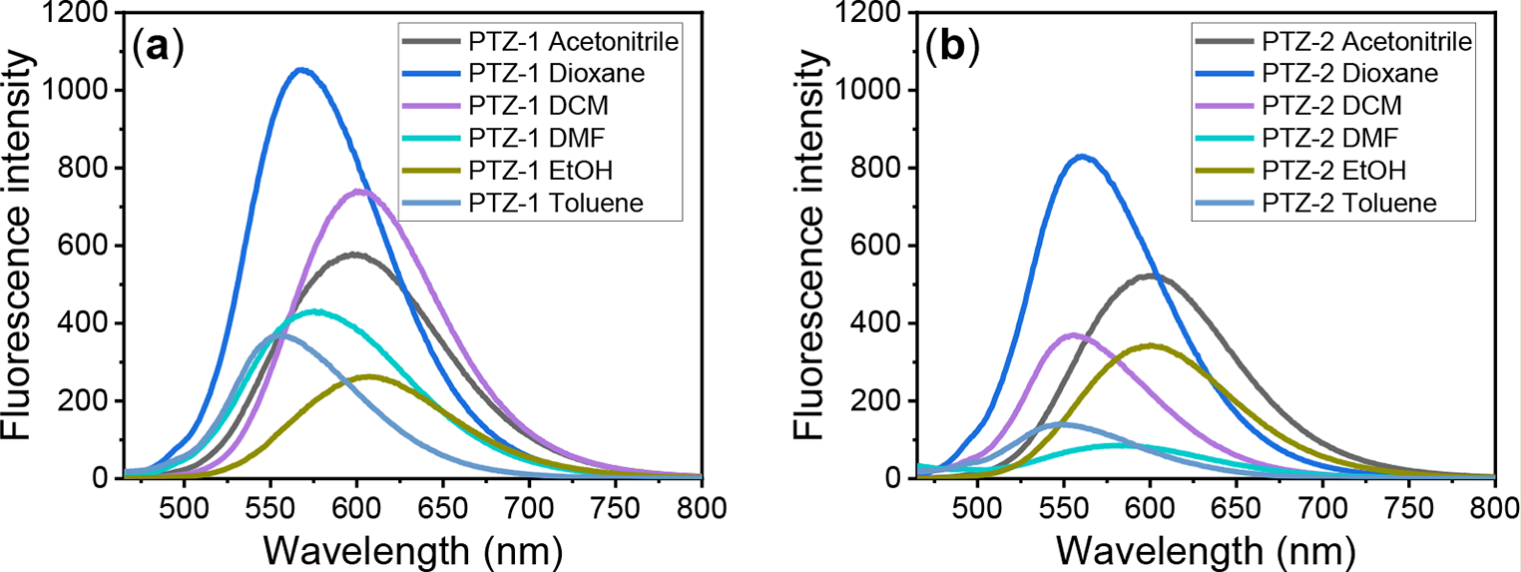
Fluorescence spectra of 10 µM (**a**) **PTZ-1** and (**b**) **PTZ-2** in different solvents

The absorption spectra of the dye solution were measured in different solvents with varying polarities, as shown in Fig. 1. No significant bathochromic or hypsochromic effect due to the polarity was observed, and both dyes absorb in the range of 391–400 nm. However, a significant emission shift to longer wavelengths was observed by increasing the polarity, as shown in Tables 1and Fig. 2. The emission spectra were recorded at 554 and 546 nm in the lowest solvent polarity, while in the highest solvent polarity, the emission maxima were recorded at 607 and 601 nm for **PTZ-1** and **PTZ-2**, respectively. The quantum yield of fluorescence increased by decreasing the solvent polarity for both **PTZ-1** and **PTZ-2**, with values of 0.62 and 0.60 for low-polarity (dioxane) solvents, and 0.20 and 0.23 for high-polarity (ethanol) solvents, respectively. The odd results observed in the case of toluene are ascribed to the low solubility of both dyes, which recorded lower quantum yield compared with DCM despite toluene having lower polarity than DCM.

**Fig. 3:**
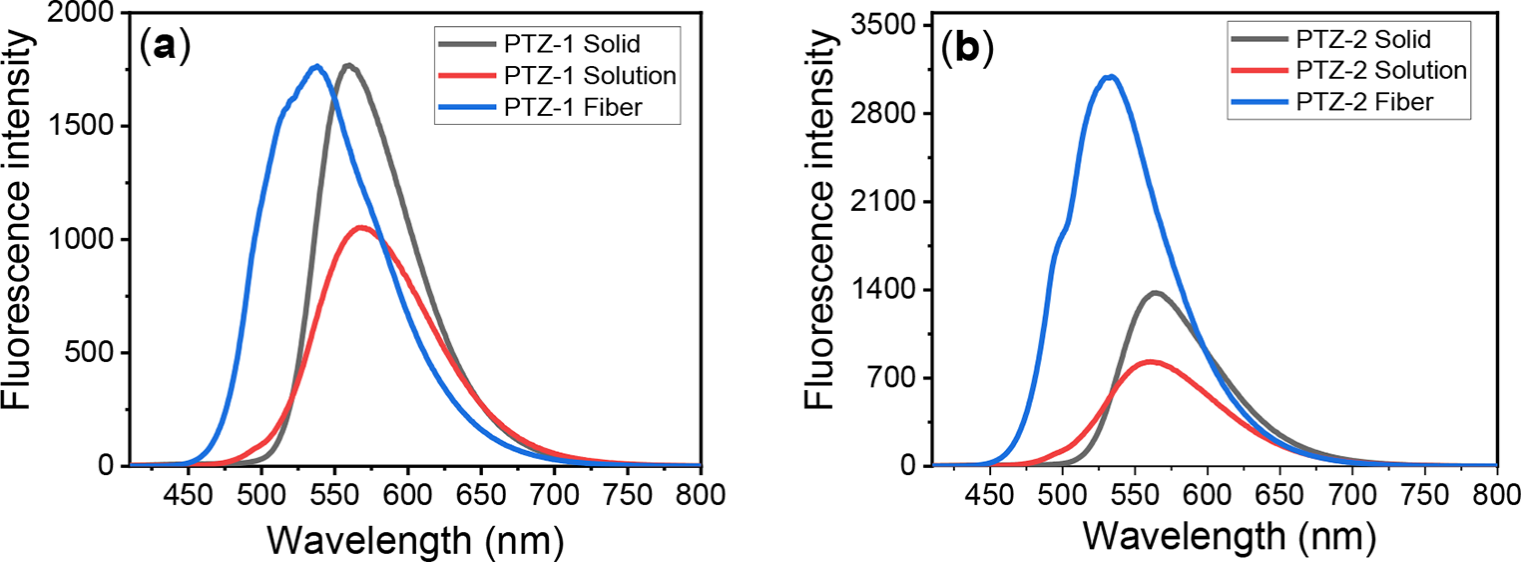
Fluorescence spectra in dioxane, solid and on dyed PET fabrics for dyes (**a**) **PTZ-1** and (**b**) **PTZ-2**

The emission was measured in polycrystalline form (powder form) for both dyes, showing very intensive emission in powder form at a shorter emission wavelength compared with the emission in solution, which was recorded at 558 and 563 nm, respectively. The Stokes shift decreased by decreasing the solvent polarity for both prepared dyes **PTZ-1** and **PTZ-2**. Moreover, these dyes showed a high Stokes shift, which reached over 200 nm in a polar protic solvent. The explanation of the large Stokes shift of the prepared dyes may be ascribed to the twisted intramolecular charge transfer (TICT) or enhanced intramolecular charge transfer (ICT) state present in push-pull (donor (D) – acceptor (A)) chromophores with large Stokes shifts. This state can be produced by solvent polarity, hydrogen bonding, or charge-transfer interactions at the locally excited state of the dye molecules [57–59]. The emission spectra were measured on the dyed fabrics using dyes **PTZ-1** and **PTZ-2**, illustrating very strong emission as presented in Fig. 3.

**Fig. 4:**
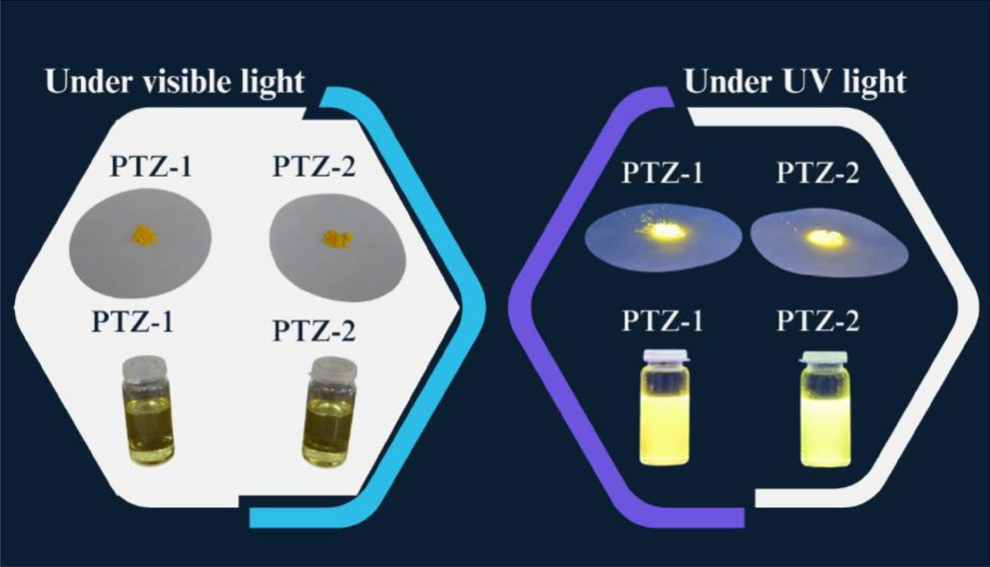
Image of the prepared dyes in solution and powder form under visible and UV light

The image of the prepared dyes in both solution and powder form under visible and UV light is illustrated in Fig. 4, showing strong emission in both forms.

### Aggregation-Induced Emission (AIE)

**Fig. 5:**
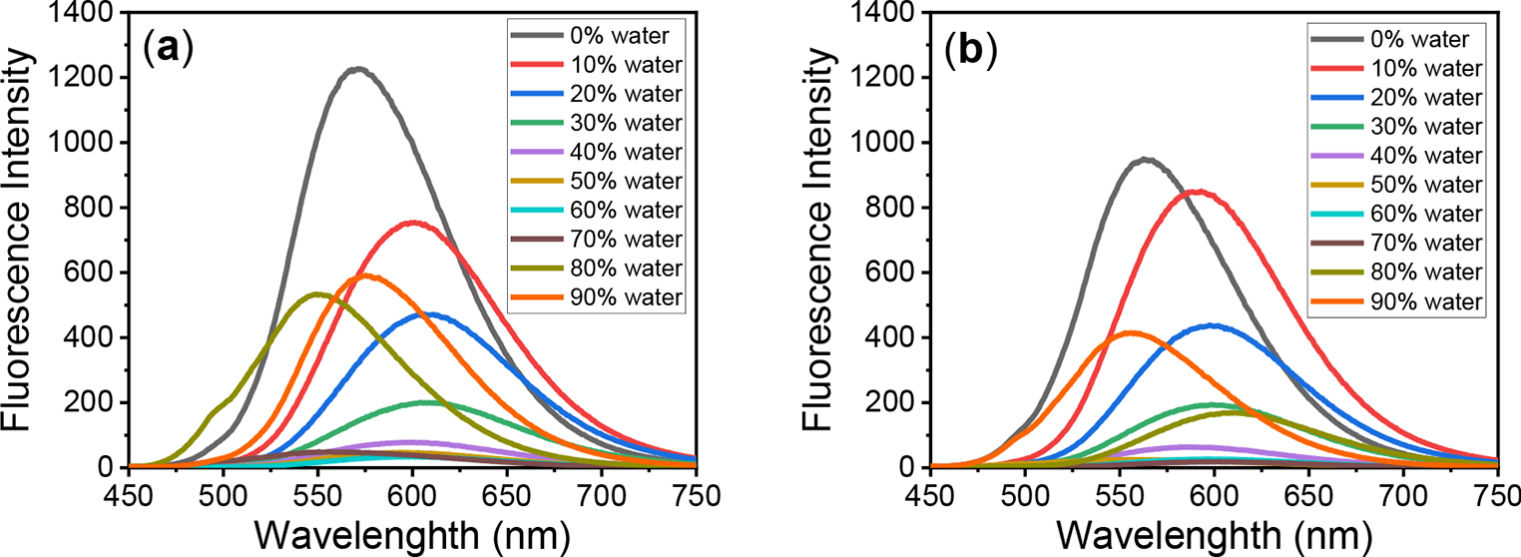
Fluorescence spectra of (**a**) **PTZ-1** and (**b**) **PTZ-2** in water-dioxane mixtures ranging from 0–90% water

**Fig. 6:**
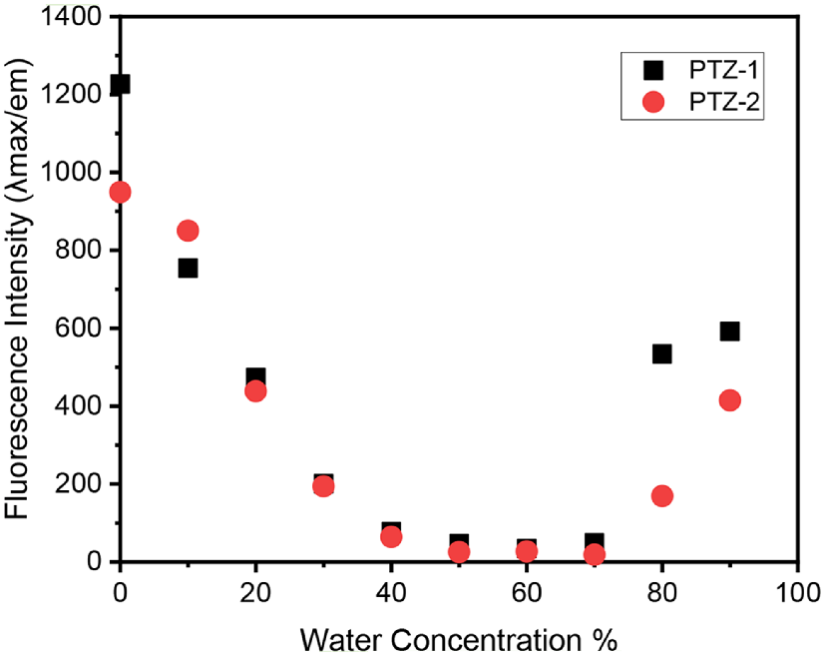
Fluorescence intensity of **PTZ-1**and **PTZ-2** in water-to-dioxane mixtures(0–90% water)

The aggregation-induced emission (AIE) of **PTZ-1** and **PTZ-2** dyes was studied by monitoring changes in fluorescent intensity at different water ratios in a dye solution in dioxane. In this system, dioxane acts as a good solvent and water behaves as a non-solvent. Dye emission intensity decreased when the water ratio is less than 70% due to quenching caused by aggregation. However, AIE occurs with an increasing water ratio greater than 70%, leading to a further increase in emission intensity. The strong polarity of the water-dioxane mixture promotes AIE and stabilizes intramolecular charge transfer. A significant shift to a longer wavelength was observed with the addition of water due to an increase in the solvent mixture polarity up to 60% water. Then, a hypsofluoric shift (shift to a shorter wavelength) was observed when the aggregation of the dye was observed, as shown in Figs. 5 and 6, and Table 2. The increase in emission due to aggregation is attributed to restricted intramolecular rotations and rigid molecular conformations of the dye structure [58].

**Table 2:** The fluorescence properties of the synthesized dyes in water-to-dioxane mixtures (0–90% water)

Water %	Dyes			
	PTZ-1		PTZ-2	
	λ_max/em_ (nm)	Fluorescence Intensity	λ_max/em_ (nm)	Fluorescence Intensity
0	571	1226	563	949
10	601	754	593	851
20	606	473	598	438
30	604	200	599	194
40	599	78	583	64
50	593	47	611	25
60	597	34	597	27
70	551	49	602	19
80	549	534	556	169
90	576	591	553	415

### The Effect of NaCl on Fluorescence Properties of PTZ Dyes

**Fig. 7:**
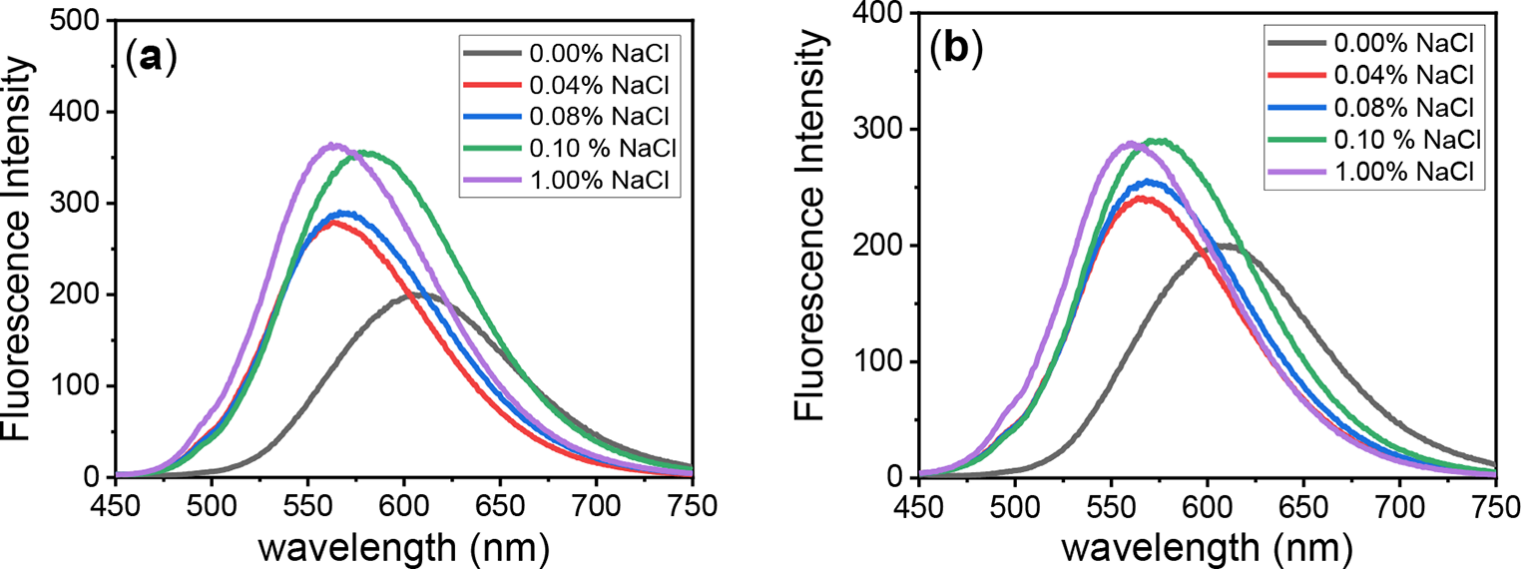
Fluorescence spectra of dyes (**a**) **PTZ-1** (**b**) **PTZ-2** 10 µmol in a Dioxane-Water 30% solution with different NaCl concentrations, ranging from 0.00–1.00%

Sodium chloride solution was used and mixed in different ratios with the dye solution in dioxane to enhance the aggregation of the dye and study the AIE by recording the emission change. The fluorescence spectra of dyes **PTZ-1** and **PTZ-2** in dioxane at various NaCl concentrations (0.00–1.00%) are shown in Fig. 7.

The graph above helps explain how various dyes react to change in ionic strength. The fluorescence intensity of **PTZ-1** and **PTZ-2** significantly increases with NaCl concentration up to 0.1% and then stabilizes. In this behavior, dye molecules aggregating in the solid state of the prepared dyes enhance emission.

Fluorescence spectra of **PTZ-1** and **PTZ-2** at various NaCl concentrations reveal their solid-state characteristics. In solid-state electronics and systems where fluorescence responsiveness to ionic concentration is critical, these dyes enhanced fluorescence intensity proportional to the dye concentration in NaCl highlights their prospective use.

**Fig. 8:**
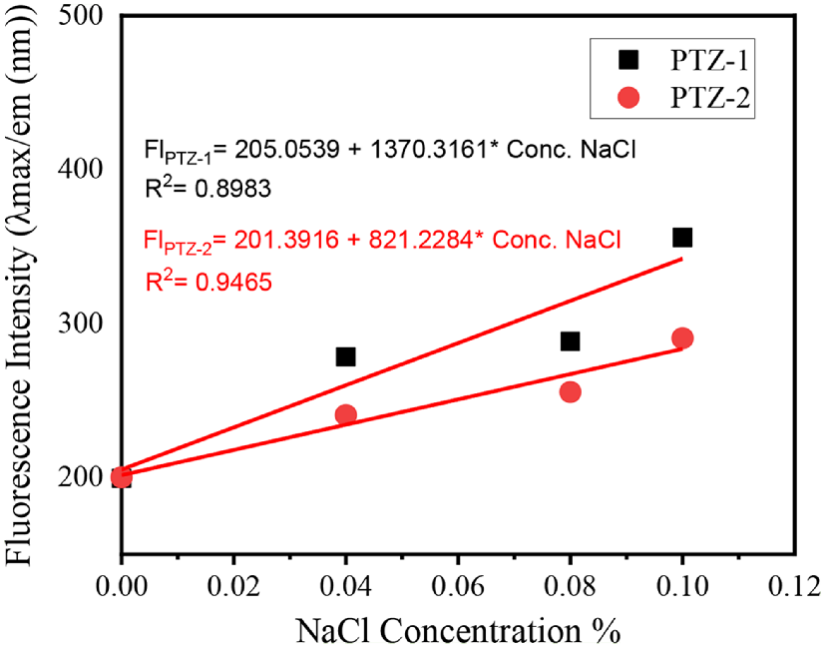
Linearity graph for the effect of sodium chloride concentration (0–1% NaCl) in the emission changes for **PTZ-1**and **PTZ-2** in dioxane mixtures

As the concentration of sodium chloride (NaCl) increases, significant fluorescence increase was recorded for both dyes **PTZ-1** and **PTZ-2** in dioxane, as shown in Fig. 8; Table 3. The linear equation presented in Fig. 8, proves the aggregation induced emission.

**Table 3:** The fluorescence properties of the synthesized dyes in NaCl solution to dioxane (0–1% NaCl)

NaCl %	Dyes			
	PTZ-1		PTZ-2	
	λ_max/em_ (nm)	Fluorescence Intensity	λ_max/em_ (nm)	Fluorescence Intensity
0	610	199	608	200
0.04	563	278	505	240
0.08	569	288	569	255
0.1	581	356	572	290
1.0	564	363	559	289

### Dyeing Application on Polyester Fabrics

**Fig. 9:**
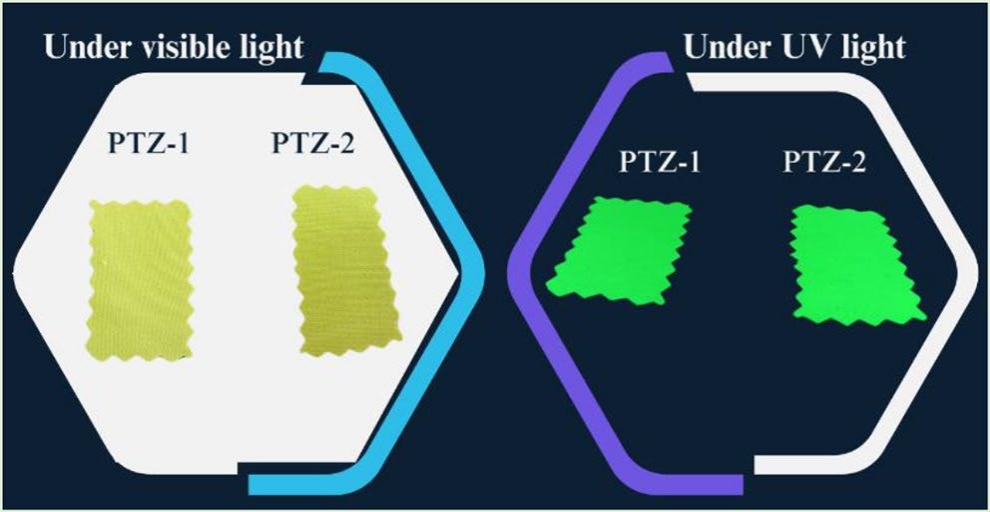
The image of the dyed polyester fabrics under visible and UV light

The dyeing application of the prepared dyes was studied on polyester fabric. The dye1 (**PTZ-1**) and dye2 (**PTZ-2**) show excellent affinity in the dyeing of PET with very high visible and bright yellow colors as shown in Fig. 9. The dye exhaustion and dye uptake were measured and summarized in Table 4 for 2% omf dye shade.

**Table 4:** The dye exhaustion and dye uptake on polyester fabrics 2% omf dye shade

Dye	K/S	%E
**PTZ-1**	7.5	92
**PTZ-2**	7.3	91

**Fig. 10:**
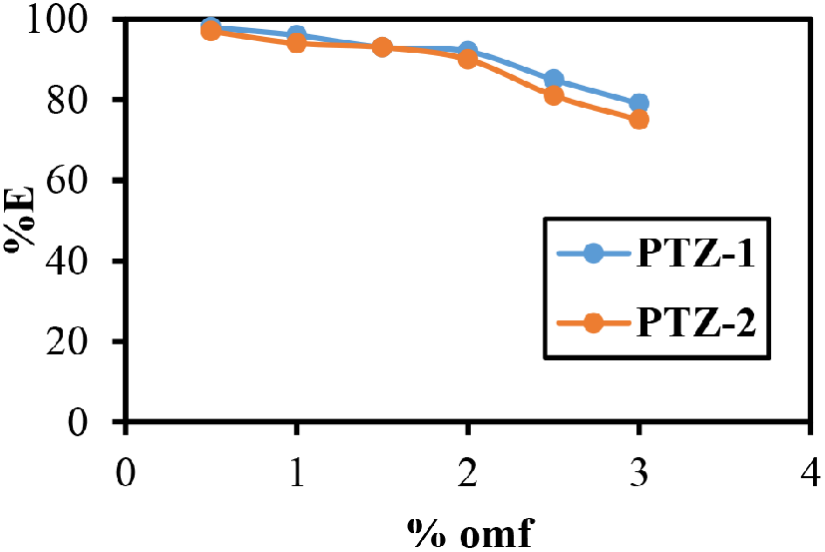
The effect of the dye concentration in %E of PET dyeing at pH 5, 130 °C

The effect of dye concentration was studied which showing very high affinity to PET fabrics and by increase the dye concentration a significant decrease in the dye exhaustion was observed, this ascribed to the possibility of the dye aggregation by increase the dye concentration as shown in Fig. 10.

The dye fastness on the dyed PET samples was studied and listed in Table 5. The dyed samples showed very good fastness against washing, light, rubbing and sublimation at 180, 220 °C. The good fastness properties of the prepared dyes to washing and rubbing is attributed to the high diffusion performance of the dye molecule into the fiber core in additional to the physical interaction between the dye molecule and polyester fiber via intermolecular hydrogen bond. The suitable small size of the dye structure and its intermolecular hydrogen bond with the fiber led to the good thermal stability of the dyed fabrics. The dyes showing good light fastness which attributed to the phenothiazine chromophore moiety.

**Table 5:** The color fastness of the dyed PET fabrics 2% omf

Dye	Washing			Rubbing		Sublimation		Light
	Alt	SC	SP	wet	dry	180 °C	220 °C	
**PTZ-1**	4–5	4–5	4–5	4–5	4–5	4–5	4–5	5
**PTZ-2**	4–5	4–5	4–5	4–5	4–5	4–5	4–5	4–5

### Antibacterial

The preventing bacterial growth using disc diffusion method of the prepared dyes powder was investigated against Gram + ve and Gram –ve bacteria such as *Staphylococcus aureus, Escherichia coli*, *Candida albicans* (Table 6). Results indicated that **PTZ-1** exhibited low antimicrobial activities against *S. aureus, E. coli* and *C. albicans* with inhibition values of 11, 9 and 12 mm, respectively. On the other hand, **PTZ-2** exhibited significant antimicrobial activities against the same test microbes with inhibition values of 17, 15 and 16 mm, respectively.

**Table 6:** The antimicrobial activity of synthesized dyes PTZ-1 and PTZ-2 against Gram + ve and Gram –ve bacteria

Dye	Clear zone		
	C. albicans	E. coli	S. aureus
PTZ-1	11	9	12
PTZ-2	17	15	16
Sulfadiazine	9	7	10
Sulfathiazole	15	13	14

The **PTZ-2** shows very good inhibition against bacterial growth compared to **PTZ-1**, which shows lower inhibitions. This result is attributed to the high affinity of **PTZ-2** due to the sulfathiazole moiety, known as an antibacterial drug compared to the sulfadiazine derivative. The antibacterial affinity of the prepared dyes **PTZ-1** and **PTZ-2** shows a nominal increase compared to the starting sulfa-drugs as presented in Table 6. The dye molecules may adhere to microbial surfaces quickly due to the sulfonamide group as well as the effect of the heterocycle in the dye molecules before entering cell membranes, rupturing them, and spilling cell inclusion bodies. Bacteria die as a result of the simultaneous breakdown of their enzyme systems.

The antibacterial affinity of the dyed PET was investigated by the colony forming unit (CFU) procedure. As expected due to the low affinity of **PTZ-1**, the dyed PET with **PTZ-1** did not show a significant effect, while the dyed PET with **PTZ-2** showed good affinity compared to **PTZ-1** as presented in Table 7.

**Table 7:** The CFU value of **PTZ-1** and **PTZ-2** against and S. aureus and E. coli

Bacteria	Sample	Absorbance at 600 nm		CFU	
		Abs. (Au)	R (%)	CFU	R (%)
*Staphylococcus aureus*	Control 1 2	2.104 0.687 0.332	- 67.35 84.22	101 25 7	- 75.25 89.52
*Escherichia coli*	Control 1 2	1.984 0.648 0.262	- 67.33 86.79	93 27 10	- 70.97 89.25

## Conclusions

Two new dyes based on phenothiazine were effectively synthesized and characterized in this research endeavor. These dyes were modified with sulfadiazine and sulfathiazole groups. The dyes exhibited significant fluorescence characteristics in both solution and their polycrystalline state, as evidenced by the 0.63 and 0.60 quantum yields for dyes **PTZ-1** and **PTZ-2** in dioxane, respectively. Their remarkable affinity and fastness on polyester fabrics with brilliant yellow hues underscore their promising prospects in the realm of high visible textile applications. Furthermore, it should be noted that the dyes demonstrated noteworthy antibacterial efficacy against both Gram + ve and Gram –ve bacteria, thus emphasizing their versatile nature. Consistent with recent developments in the domain of emissive materials, this study’s findings mirror the progress made on phenothiazine-based D–π–A dyes, which exhibited emissive solvatochromism and AIE activity. The results obtained from this study make a valuable contribution to the expanding field of research concerning functional dyes and their potential uses in diverse sectors, such as antimicrobial and optoelectronic therapies.

## Data Availability

No datasets were generated or analysed during the current study.

## References

[1] Pérez–Gutiérrez E, Percino MJ, Bernal W, Cerón M, Ceballos P, Rivadeneyra MS, Siegler MA, Thamotharan S (2021, February) Fluorescence tuning with a single dye embedded in a polymer matrix and its application on multicolor OLEDs. Dyes Pigm 186:108979. 10.1016/j.dyepig.2020.108979

[2] Qu W, Gao Z, Li W, Fan X, Shi Y, Miao Y, Wu Z, Huang J, Wang H, Wei B (2022, March) Carbazole/triazine based host materials for high-performance green PhOLEDs. Dyes Pigm 199:110086. 10.1016/j.dyepig.2022.110086

[3] Deng X, Liu S, Sun Y, Zhong D, Jia D, Yang X, Su B, Sun Y, Zhou G, Jiao B, Wu Z (2023) January). 9-Phenyl-9-phosphafluorene oxide based organic ligands synthesized via successive SNAr reactions and their symmetric phosphorescent ir(III) complexes for highly efficient solution-processed OLEDs. Dyes Pigm 209:110885. 10.1016/j.dyepig.2022.110885

[4] Coban MB (2023) January). A new 3D HoIII-organic framework constructed from 1,3,5-tris(4-carboxyphenyl)benzene and 1,10-phenanthroline: Crystal structure, morphological and solid state luminescence properties. J Solid State Chem 317:123651. 10.1016/j.jssc.2022.123651

[5] Mohamed MB, El-Sedik MS, Youssef YA, Mohamed NA, Aysha TS (2022, December) New stilbene-biscarbothioamide based colorimetric chemosensor and turn on fluorescent probe for recognition of Hg2 + cation. J Photochem Photobiol A 433:114206. 10.1016/j.jphotochem.2022.114206

[6] Aysha T, Zain M, Arief M, Youssef Y (2022, February) Alkali-stable solid state fluorescent pyrazolo/pyrrolinone disperse dyes: synthesis and application for dyeing polyester fabric. J Mol Struct 1249:131623. 10.1016/j.molstruc.2021.131623

[7] El-Sedik M, Aysha T, Youssef Y (2016, December) Synthesis, photophysical properties, and application of optical brighteners based on stilbene‐oxadiazole derivatives. Color Technol 133(2):122–127. 10.1111/cote.12258

[8] Elmorsi TM, Aysha TS, Sheier MB, Bedair AH (2017) May 24). Synthesis, Kinetics, and Equilibrium Study of highly sensitive colorimetric chemosensor for monitoring of copper ions based on Benzo[f]fluorescein dye derivatives. Z Für Anorganische Und Allgemeine Chemie 643(13):811–818. 10.1002/zaac.201700112

[9] Dhouib S, Lallam A, Sakli F (2006, April) Study of Dyeing Behavior of Polyester fibers with disperse dyes. Text Res J 76(4):271–280. 10.1177/0040517506061243

[10] Aysha T, Zain M, Arief M, Youssef Y (2019, August) Synthesis and spectral properties of new fluorescent hydrazone disperse dyes and their dyeing application on polyester fabrics. Heliyon 5(8):e02358. 10.1016/j.heliyon.2019.e02358 10.1016/j.heliyon.2019.e02358 PMC6716976 31485538

[11] Synthesis of biscoumarin bifunctional reactive fluorescent whitening agents and their application on nylon-6 fabric (2022) March 3). Indian J Fibre Text Res 46(4). 10.56042/ijftr.v46i4.43262

[12] Chathoth AM, Subba Rao AN, Nair S, Nagarajappa GB, Pandey KK (2023) May 2). Luminescent transparent wood from a woody cellulosic template treated with an optical brightener. J Appl Polym Sci 140(28). 10.1002/app.54028

[13] Eldessouki M, Eldessouki M, Aysha T, Ratičáková M, Šašková J, Padil VV, Ibrahim M, Černík M (2017) October 31). Structural parameters of functional membranes for integration in Smart Wearable materials. Fibres Textiles East Europe 25(0):73–78. 10.5604/01.3001.0010.4631

[14] Aysha T, El-Sedik M, Mashaly HM, El‐Apasery MA, Machalický O, Hrdina R (2015) July 8). Synthesis, characterisation, and applications of isoindigo/Pechmann dye heteroanalogue hybrid dyes on polyester fabric. Color Technol 131(4):333–341. 10.1111/cote.12161

[15] Traven VF, Cheptsov DA, Solovjova NP, Chibisova TA, Voronov II, Dolotov SM, Ivanov IV (2017) November). Photoinduced formation of the laser dye coumarin 6 from its dihydro derivatives. Dyes Pigm 146:159–168. 10.1016/j.dyepig.2017.07.001

[16] Dinastiya EM, Verbitskiy EV, Gadirov RM, Samsonova LG, Degtyarenko KM, Grigoryev DV, Kurtcevich AE, Solodova TA, Tel’minov EN, Rusinov GL, Chupakhin ON, Charushin VN (2021) March). Investigation of 4,6-di(hetero)aryl-substituted pyrimidines as emitters for non-doped OLED and laser dyes. J Photochem Photobiol A 408:113089. 10.1016/j.jphotochem.2020.113089

[17] Cheng W, Chen H, Liu C, Ji C, Ma G, Yin M (2020) July 30). Functional organic dyes for health-related applications. VIEW 1(4). 10.1002/viw.20200055

[18] Krishnamoorthy K, Premanathan M, Veerapandian M, Jae Kim S (2014), July 17 Nanostructured molybdenum oxide-based antibacterial paint: effective growth inhibition of various pathogenic bacteria. *Nanotechnology*, *25*(31), 315101. 10.1088/0957-4484/25/31/315101 10.1088/0957-4484/25/31/315101 25030310

[19] Hong Y, Lam JWY, Tang BZ (2011) Aggregation-induced emission. Chem Soc Rev 40(11):5361. 10.1039/c1cs15113d 21799992 10.1039/c1cs15113d

[20] Mei J, Hong Y, Lam JWY, Qin A, Tang Y, Tang BZ (2014) June 30). Aggregation-Induced Emission: the whole is more brilliant than the parts. Adv Mater 26(31):5429–5479. 10.1002/adma.201401356 24975272 10.1002/adma.201401356

[21] Zhao Z, Zhang H, Lam JWY, Tang BZ (2020) May 14). Aggregation-Induced Emission: New Vistas at the aggregate level. Angew Chem Int Ed 59(25):9888–9907. 10.1002/anie.201916729 10.1002/anie.201916729 32048428

[22] Chen Y, Lam JWY, Kwok RTK, Liu B, Tang BZ (2019) Aggregation-induced emission: fundamental understanding and future developments. Mater Horiz 6(3):428–433. 10.1039/c8mh01331d

[23] Wu W, Liu B (2020), August 31 Aggregation-induced emission: challenges and opportunities. *National Science Review*, *8*(6). 10.1093/nsr/nwaa222 10.1093/nsr/nwaa222 PMC8288179 34691662

[24] Liu B, Tang BZ (2020) May 25). Aggregation-Induced Emission: more is different. Angew Chem Int Ed 59(25):9788–9789. 10.1002/anie.202005345 10.1002/anie.202005345 32449572

[25] Yu Y, Jia H, Liu Y, Zhang L, Feng G, Tang BZ (2022) December 31). Recent progress in type I Aggregation-Induced Emission Photosensitizers for photodynamic therapy. Molecules 28(1):332. 10.3390/molecules28010332 36615526 10.3390/molecules28010332 PMC9822449

[26] Jiang Z, Zhang Q, Kong Z, Qiao R, Liu Z, Song L, Zhu S, Liu R, Zhu H, March (2024) Aggregation-induced phosphorescence emission-active heteroleptic ir(III) complexes: synthesis, photophysics, and latent fingerprint detection applications. Dyes Pigm, 222, 111837. 10.1016/j.dyepig.2023.111837

[27] Wang J, Yang Y, Jiang C, He M, Yao C, Zhang J (2022) Ultrapure deep-blue aggregation-induced emission and thermally activated delayed fluorescence emitters for efficient OLEDs with CIEy < 0.1 and low efficiency roll-offs. J Mater Chem C 10(8):3163–3171. 10.1039/d1tc05497j

[28] Cai X, Liu B (2020), May 7 Aggregation-Induced Emission: Recent Advances in Materials and Biomedical Applications. *Angewandte Chemie International Edition*, *59*(25), 9868–9886. 10.1002/anie.202000845 10.1002/anie.202000845 32128951

[29] Hong Y, Lam JWY, Tang BZ (2009) Aggregation-induced emission: phenomenon, mechanism and applications. Chem Commun 29:4332. 10.1039/b904665h 10.1039/b904665h 19597589

[30] Manivannan R, Han M, Patra SK, Prabakaran K, Kim W, Lim SK, Oh J, Son YA (2024, January) Effect of thermal, mechanical and photophysical properties of high emissive photoluminescence organic pigments. Dyes Pigm 221:111785. 10.1016/j.dyepig.2023.111785

[31] Rout Y, Montanari C, Pasciucco E, Misra R, Carlotti B (2021), June 23 Tuning the Fluorescence and the Intramolecular Charge Transfer of Phenothiazine Dipolar and Quadrupolar Derivatives by Oxygen Functionalization. *Journal of the American Chemical Society*, *143*(26), 9933–9943. 10.1021/jacs.1c04173 10.1021/jacs.1c04173 PMC8297855 34161725

[32] Li R, Wang S, Li Q, Lan H, Xiao S, Li Y, Tan R, Yi T (2017) February). A fluorescent non-conventional organogelator with gelation-assisted piezochromic and fluoride-sensing properties. Dyes Pigm 137:111–116. 10.1016/j.dyepig.2016.10.004

[33] Li D, Yu J, Xu R (2011) Mesoporous silica functionalized with an AIE luminogen for drug delivery. Chem Commun 47(39):11077. 10.1039/c1cc14064g 10.1039/c1cc14064g 21897920

[34] TamilSelvan S, Prakasam A, Venkatesh G, Kamal C, Sheena Mary Y, Banu P, Vennila S, P., Mary S, Y (2020) November 26). Synthesis, spectral characterizations, molecular geometries and electronic properties of phenothiazine based organic dyes for dye-sensitized solar cells. Z fÃ¼r Phys Chem 235(10):1355–1380. 10.1515/zpch-2020-1732

[35] Al-Ghamdi SN, Al-Ghamdi HA, El-Shishtawy RM, Asiri AM (2021) October). Advances in phenothiazine and phenoxazine-based electron donors for organic dye-sensitized solar cells. Dyes Pigm 194:109638. 10.1016/j.dyepig.2021.109638

[36] Ouared I, Rekis M, Trari M (2021, June) Phenothiazine based organic dyes for dye sensitized solar cells: a theoretical study on the role of π-spacer. Dyes Pigm 190:109330. 10.1016/j.dyepig.2021.109330

[37] Li S, He J, Jiang H, Mei S, Hu Z, Kong X, Yang M, Wu Y, Zhang S, Tan H (2021) March 4). Comparative studies on the structure–performance relationships of Phenothiazine-based Organic dyes for Dye-Sensitized Solar cells. ACS Omega 6(10):6817–6823. 10.1021/acsomega.0c05887 33748595 10.1021/acsomega.0c05887 PMC7970489

[38] Nagarajan B, Elumalai CDA, Chandran R, S., Raghavachari D (2021, January) Naphthalimide-Phenothiazine based A’-π-D-π-A featured organic dyes for dye sensitized solar cell applications. J Photochem Photobiol A 404:112820. 10.1016/j.jphotochem.2020.112820

[39] Găină L, Gal E, Mătărângă-Popa L, Porumb D, Nicolescu A, Cristea C, Silaghi-Dumitrescu L (2012, March) Synthesis, structural investigations, and DFT calculations on novel 3-(1,3-dioxan-2-yl)-10-methyl-10H-phenothiazine derivatives with fluorescence properties. Tetrahedron 68(11):2465–2470. 10.1016/j.tet.2012.01.068

[40] Bieliauskas A, Martynaitis V, Getautis V, Malinauskas T, Jankauskas V, Kamarauskas E, Holzer W, Šačkus A (2012, May) Synthesis of electroactive hydrazones derived from 3-(10-alkyl-10H-phenothiazin-3-yl)-2-propenals and their corresponding 3,3′-bispropenals. Tetrahedron 68(18):3552–3559. 10.1016/j.tet.2012.03.010

[41] Golzar Hossain G (2013, July) Synthesis and characterisation of cobalt complex of sulfathiazole with acetic acid. J Saudi Chem Soc 17(3):253–257. 10.1016/j.jscs.2011.04.002

[42] Bellú S, Hure E, Trapé M, Rizzotto M, Sutich E, Sigrist M, Moreno V (2003, March) The interaction between mercury(II) and sulfathiazole. Quím Nova 26(2):188–192. 10.1590/s0100-40422003000200008

[43] Edozie OI, Godday OJ, Chijioke AK, Uchenna IO, Chigozie NF (2020), April 24 Synthesis, characterization and molecular docking studies of Co(II) metal complex of sulfathiazole. *Bulletin of the Chemical Society of Ethiopia*, *34*(1), 83–92. 10.4314/bcse.v34i1.8

[44] Morais D, Guedes R, Lopes M (2016) June 21). Antimicrobial approaches for textiles: from research to market. Materials 9(6):498. 10.3390/ma9060498 28773619 10.3390/ma9060498 PMC5456784

[45] Yang X, Chung E, Johnston I, Ren G, Cheong YK (2021) May 15). Exploitation of Antimicrobial nanoparticles and their applications in Biomedical Engineering. Appl Sci 11(10):4520. 10.3390/app11104520

[46] Park JH, Cho NS, Jung YK, Cho HJ, Shim HK, Kim H, Lee YS (2007, April) Polymeric light emitting properties and structural relationships of fluorene-based conjugated copolymers containing various hole transporting derivatives. Org Electron 8(2–3):272–285. 10.1016/j.orgel.2006.08.002

[47] Wang H, Xu W, Zhang B, Xiao W, Wu H (2008) November 26). 10-Hexyl-10H-phenothiazine-3-carbaldehyde. Acta Crystallogr Sect E Struct Rep Online 64(12):o2458–o2458. 10.1107/s1600536808038890 21581426 10.1107/S1600536808038890 PMC2960062

[48] Jyothi NR, Farook M, Madhuri N, J., Gowthami K (2020) December 30). Synthesis, characterization of copper complexes of9H-Carbazole-3-carbaldehyde-4-phenylthiosemicarbazone, 10-Hexyl-10-H-phenothiazine-3-carbaldehyde-4-phenylthio semicarbazone and 2-Thiophenecarboxaldehyde-4-methyl- thiosemicarbazone and anti-bacterial activity studies of ligands and complexes. Orient J Chem 36(6):1119–1119. 10.13005/ojc/360615

[49] Hauck M, Schönhaber J, Zucchero AJ, Hardcastle KI, Müller TJJ, Bunz UHF (2007), August 10 Phenothiazine Cruciforms: Synthesis and Metallochromic Properties. *The Journal of Organic Chemistry*, *72*(18), 6714–6725. 10.1021/jo070922l 10.1021/jo070922l 17691738

[50] Sun W, Sun P, Zhu Y, Wang H, Li S, Wu J, Tian Y (2015, April) Syntheses, crystal structures, nonlinear optical properties and cis-trans isomerization of functionalized sulfur-terminal [Zn(II) and cd (II)] complexes based on phenothiazine-2,2′:6′,2″-terpyridine conjugated ligands. Dyes Pigm 115:110–119. 10.1016/j.dyepig.2014.12.011

[51] Eaton DF (1988), January 1 Reference materials for fluorescence measurement. *Pure and Applied Chemistry*, *60*(7), 1107–1114. 10.1351/pac198860071107

[52] Gupta D, Khare SK, Laha A (2004, July) Antimicrobial properties of natural dyes against Gram-negative bacteria. Color Technol 120(4):167–171. 10.1111/j.1478-4408.2004.tb00224.x

[53] Judd DB, Nickerson D (1975), January 1 Relation between Munsell and Swedish Natural Color System scales. *Journal of the Optical Society of America*, *65*(1), 85. 10.1364/josa.65.000085 10.1364/josa.65.000085 1110426

[54] Hall AJ (1996, May) Colour fastness standards for textiles and leather: from BS 1006 to BS EN ISO 105. J Soc Dyers Colour 112(5–6):144–145. 10.1111/j.1478-4408.1996.tb01804.x

[55] Cui G, Luo MR, Rigg B, Butterworth M, Dakin J (2004, September) Grading textile fastness. Part 3: development of a new fastness formula for assessing change in colour. Color Technol 120(5):226–230. 10.1111/j.1478-4408.2004.tb00122.x

[56] ISO 105-B02 (2013) :2013 textiles: tests for colour fastness. Part B02: Colour fastness to artificial light: Xenon arc fading lamp test. ISO, Basel

[57] Muruganantham S, Velmurugan G, Jesuraj J, Hafeez H, Ryu SY, Venuvanalingam P, Renganathan R (2019) Impact of tunable 2-(1H-indol-3-yl)acetonitrile based fluorophores towards optical, thermal and electroluminescence properties. RSC Adv 9(25):14544–14557. 10.1039/c8ra10448d 35519310 10.1039/c8ra10448d PMC9064231

[58] Al Sharif OF, Nhari LM, El-Shishtawy RM, Zayed MEM, Asiri AM (2022) AIE and reversible mechanofluorochromism characteristics of new imidazole-based donor–π–acceptor dyes. RSC Adv 12(30):19270–19283. 10.1039/d2ra01466a 35865558 10.1039/d2ra01466a PMC9248369

[59] Sachdeva T, Milton MD (2020) November). Fluorescent dyes for moisture detection in organic solvents: push-pull based phenothiazine aldehydes with large Stokes shifts. J Photochem Photobiol A 402:112804. 10.1016/j.jphotochem.2020.112804

