# Correction to: Synthesis of New D–π–A Phenothiazine-Based Fluorescent Dyes: Aggregation Induced Emission and Antibacterial Activity

**DOI:** 10.1007/s10895-024-03757-y

**Published:** 2024-05-08

**Authors:** Mervat S. El-Sedik, Mahmoud Basseem I. Mohamed, Mohamed S. Abdel-Aziz, Tarek S. Aysha

**Affiliations:** 1 Dyeing, Printing and Textile Auxiliaries Department, Textile Research and Technology Institute, National Research Centre, 33 EL Buhouth St, Dokki, Giza 12622 Egypt; 2 Chemistry Department, Faculty of Science, Al-Azhar University, P.O. 11884, Nasr City, Cairo Egypt; 3 Microbial Chemistry Department, Biotechnology Research Institute, National Research Centre, 33 EL Buhouth St, Dokki, Giza 12622 Egypt

**Correction to: Journal of Fluorescence**

https://doi.org/10.1007/s10895-024-03708-7

The original version of this article unfortunately contained a mistake in one of the authors’ names. The second author should be corrected from “Mahmoud BasseemI. Mohamed” to “Mahmoud Basseem I. Mohamed”. It should be written with a space between ‘Basseem’ and ‘I’.

The original article has been corrected.

